# Case report: Synchronous prostate cancer and renal cell carcinoma with prostate cancer-origin metastases to adrenal and renal hilar lymph nodes

**DOI:** 10.3389/fonc.2024.1412067

**Published:** 2024-10-07

**Authors:** Yaowen Zhang, Junru Chen, Lijing Xu, Xu Hu, Hao Zeng, Zhenhua Liu

**Affiliations:** ^1^ Department of Urology, Institute of Urology, West China Hospital, Sichuan University, Chengdu, China; ^2^ Department of Urology, West China Xiamen Hospital, Sichuan University, Xiamen, China

**Keywords:** multiple primary cancers, renal cell carcinoma, prostate cancer, metastatic tumor, adrenal metastasis, lymph node metastasis, surgical diagnostic procedures

## Abstract

**Background:**

Synchronous occurrence of prostate cancer (PCa) and renal cell carcinoma (RCC) is uncommon. RCC has a higher tendency to metastasize to the adrenal glands, renal hilar, and retroperitoneal lymph nodes compared to PCa. To date, there are no documented cases existing where metastatic tumors in these regions, observed in patients concurrently with PCa and RCC, have originated from the PCa rather than the RCC.

**Case presentation:**

In this case report, we described a 67-year-old male presented with dysuria for two months and left lower extremity edema for three days. Percutaneous biopsies revealed synchronous primary RCC and PCa. However, the origin of the metastatic tumors, especially those involving the adrenal glands, renal hilum, and retroperitoneal regions, remained undetermined. Subsequent surgical procedures identified that the metastatic lesions originated from the PCa, while the RCC was localized. Ultimately, the patient with metastatic hormone-sensitive prostate cancer (mHSPC) received combination therapy with rezvilutamide and goserelin, which resulted in a satisfactory treatment response.

**Conclusion:**

In patients with concurrent PCa and RCC, metastatic lesions in the adrenal glands, renal hilar, and retroperitoneal lymph nodes may also originate from the PCa. Accurate identification of the primary tumor and proper staging are critical for the appropriate management of patients with multiple primary malignancies with concurrent metastases.

## 1 Introduction

Prostate cancer (PCa) and renal cell carcinoma (RCC) are common tumors of the urinary system, with respective incidence rates of 7.3% and 2.2% in overall tumor landscape (1). However, the synchronous occurrence of RCC and PCa is rare (2). Most of the previous studies only reported the use of combined radical prostatectomy and partial nephrectomy as a treatment for synchronous, localized PCa and RCC (3). However, the metastatic patterns and treatment approaches for patients with coexisting PCa and RCC with metastases remain elusive owing to a lack of research. In patients with PCa, the predominant metastatic sites include the bone, lymph node, and liver (4), while RCC predominantly involves the lung, lymph node, and bone (5). In PCa, the metastatic potential to the kidney or adrenal gland is approximately 1.0%, with retroperitoneal lymph node involvement occurring in about 1.8% of cases (4). In RCC, the incidence of adrenal gland metastasis ranges from 6% to 10% (5), and the incidence of lymph node metastasis is 21.8%, with 6.8% occurring in the retroperitoneum (6). Notably, RCC exhibits a markedly higher frequency of retroperitoneal lymph node and adrenal gland metastases compared to PCa. Here, we present a case of an elderly male patient presenting with concurrent RCC and PCa. Notably, all metastatic lesions, including those in the adrenal gland and the renal hilar region, were determined to have originated from the PCa. To our knowledge, this unique coexistence has not been documented in prior reports.

## 2 Case presentation

### 2.1 Medical history and examinations

**Figure 1:**
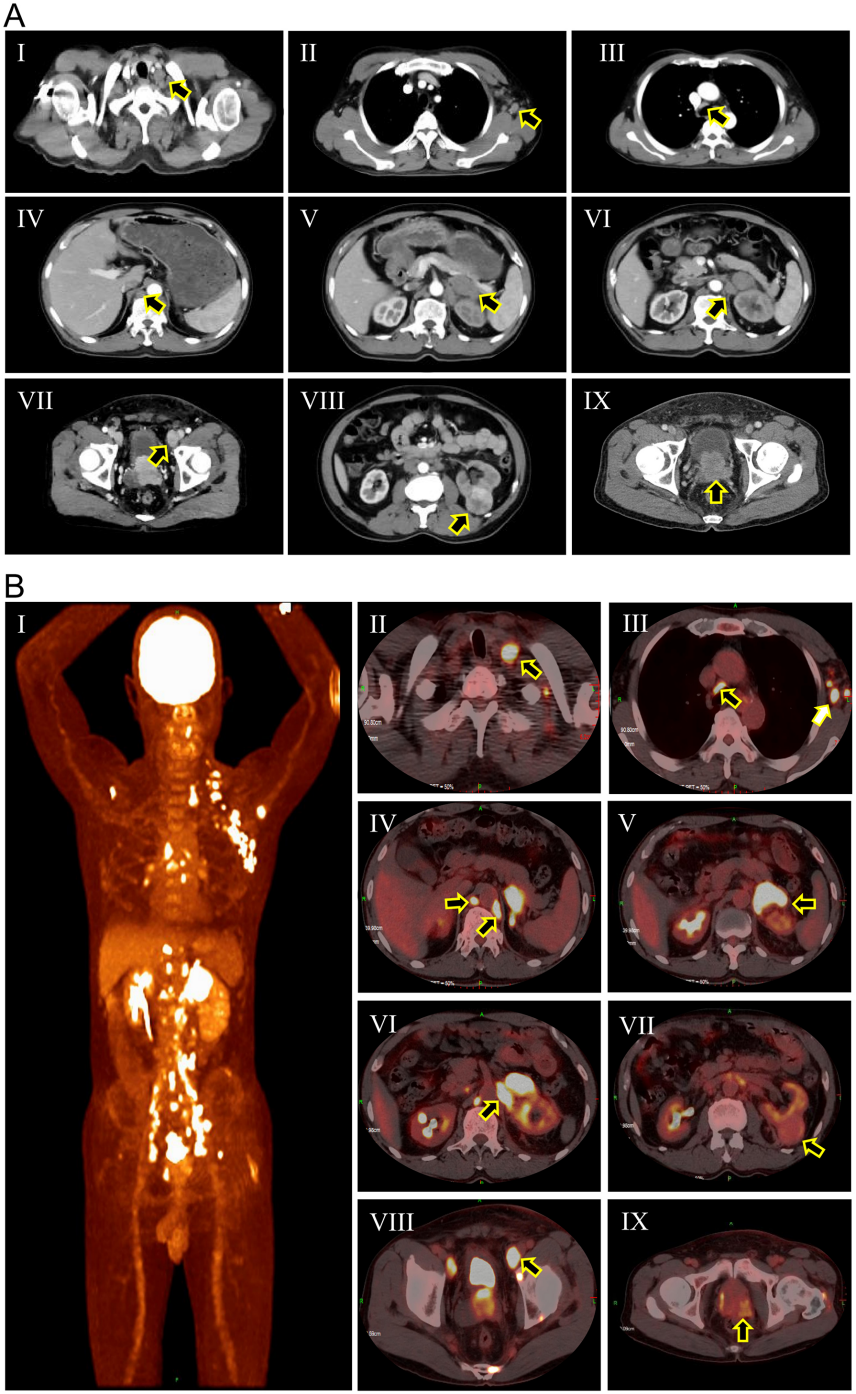
Computed tomography scan **(A)** and ^18^F-FDG PET/CT scan **(B)** of the patient at initial diagnosis. (A-I) Cervical lymph nodes (black arrow); (A-II) Axillary lymph nodes (black arrow); (A-III) Mediastinal lymph nodes (black arrow); (A-IV) Retroperitoneal lymph nodes (black arrow); (A-V) Adrenal metastasis (black arrow); (A-VI) Renal hilar lymph nodes (black arrow); (A-VII) External iliac lymph nodes (black arrow); (A-VIII) Renal mass (black arrow); (A-IX) Enlarged prostate; (B-I) Overview of pathological uptake; (B-II) Cervical lymph nodes (black arrow); (B-III) Axillary lymph nodes (white arrow) and mediastinal lymph nodes (black arrow); (B-IV) Retroperitoneal lymph nodes (black arrows). (B-V) Adrenal metastasis (black arrow); (B-VI) Renal hilar lymph nodes (black arrow); (B-VII) Renal mass (black arrow); (B-VIII) External iliac lymph nodes (black arrow); (B-IX) Prostatic mass (black arrow).

The patient was a 67-year-old male who presented with a two-month history of dysuria and a three-day history of edema in the left lower extremity. Upon admission, computed tomography (CT) scan (**Figure 1A**) revealed a 4.0 × 3.8 cm mass in the left kidney and a 4.8 × 4.0 cm mass in the left adrenal gland with irregular enhancement. Concurrently, there was evidence of increased and enlarged lymph nodes throughout multiple sites in the body, as well as irregular enhancement of the prostate and bilateral seminal vesicles. Subsequently, the ^18^F-FDG PET/CT scan (**Figure 1B**) identified areas of intense uptake in the seminal vesicles, prostate, left adrenal gland, skeletal regions, and lymph nodes of the cervical, axillary, mediastinal, and abdominal areas, which were indicative of malignancies. However, the precise location of the primary tumor could not be definitively identified. Additionally, the uptake level of the left renal mass was relatively lower than those of the aforementioned lesions but higher than that of normal tissues, suggesting that it might be a primary RCC. The digital rectal examination (DRE) revealed a firm, enlarged prostate with an indurated nodule. The patient’s prostate-specific antigen (PSA) level was 531 ng/ml, with no detected abnormalities in adrenal hormones. Physicians initially considered that the patient might have metastatic PCa (staged as T_3b_N_1_M_1b_), concurrently with RCC (staged as T_4_N_1_M_x_) that had metastasized to the adrenal gland and retroperitoneal lymph nodes.

### 2.2 Investigations

To further clarify the original tumors of this patient, we performed an ultrasound-guided percutaneous biopsy of the left renal mass and an ultrasound-guided transperineal biopsy of the prostate. The immunohistochemical results are summarized in **Table 1**, the pathologic diagnoses were primary clear cell renal cell carcinoma (ccRCC) and primary adenocarcinoma of prostate with a Gleason score of 4 + 4 = 8. Subsequently, the patient underwent a left nephrectomy, left adrenalectomy, and lymph node dissection of the left renal hilum and retroperitoneum region (**Supplementary Figure 1**). The immunohistochemical results of the resected specimens are summarized in **Table 1**. The renal mass was further confirmed as ccRCC with an International Society of Urological Pathology (ISUP) grade of 3, while the lesions of the adrenal glands, renal hilar lymph nodes and retroperitoneal lymph nodes were originated from PCa. The RCC was staged as T_1_N_0_M_0_, while the stage of PCa was determined to be T_3b_N_1_M_1c_.

**Table 1:** Immunohistochemical results of the needle biopsy and resected specimens.

Antigens (markers)	Needle biopsy		Resected specimen		
	Renal mass	Prostate	Renal mass	Adrenal mass/ Retroperitoneal lymph nodes	Renal hilar lymph nodes
PAX-8	+	–	+	–	–
NKX3.1	NA	+	NA	+	+
CD10	+	NA	+	NA	NA
CA IX	+	NA	+	NA	NA
CK7	–	NA	–	NA	NA
AMACR	NA	+	NA	NA	NA
P63	NA	–	NA	NA	NA
PSA	NA	+	NA	NA	NA
PSMA	NA	+	NA	+	NA
CgA	NA	–	NA	+, 5%	NA
Syn	NA	NA	NA	–	NA
SF-1	NA	NA	NA	–	NA
TTF-1	NA	NA	NA	–	NA

NA, not available; PAX-8, paired box gene 8; NKX3.1, NK3 homeobox 1; CD10, cluster of differentiation 10; CA IX, carbonic anhydrase IX; CK7, cytokeratin 7; AMACR, alpha-methylacyl-CoA racemase; HCK, hematopoietic cell kinase; PSA, prostate-specific antigen; PSMA, prostate-specific membrane antigen; CgA, chromogranin A; Syn, synaptophysin; SF-1, steroidogenic factor 1; TTF-1, thyroid transcription factor. +, the result of immunohistochemical staining is positive; -, the result of immunohistochemical staining is negative.

### 2.3 Treatment and outcome

**Figure 2:**
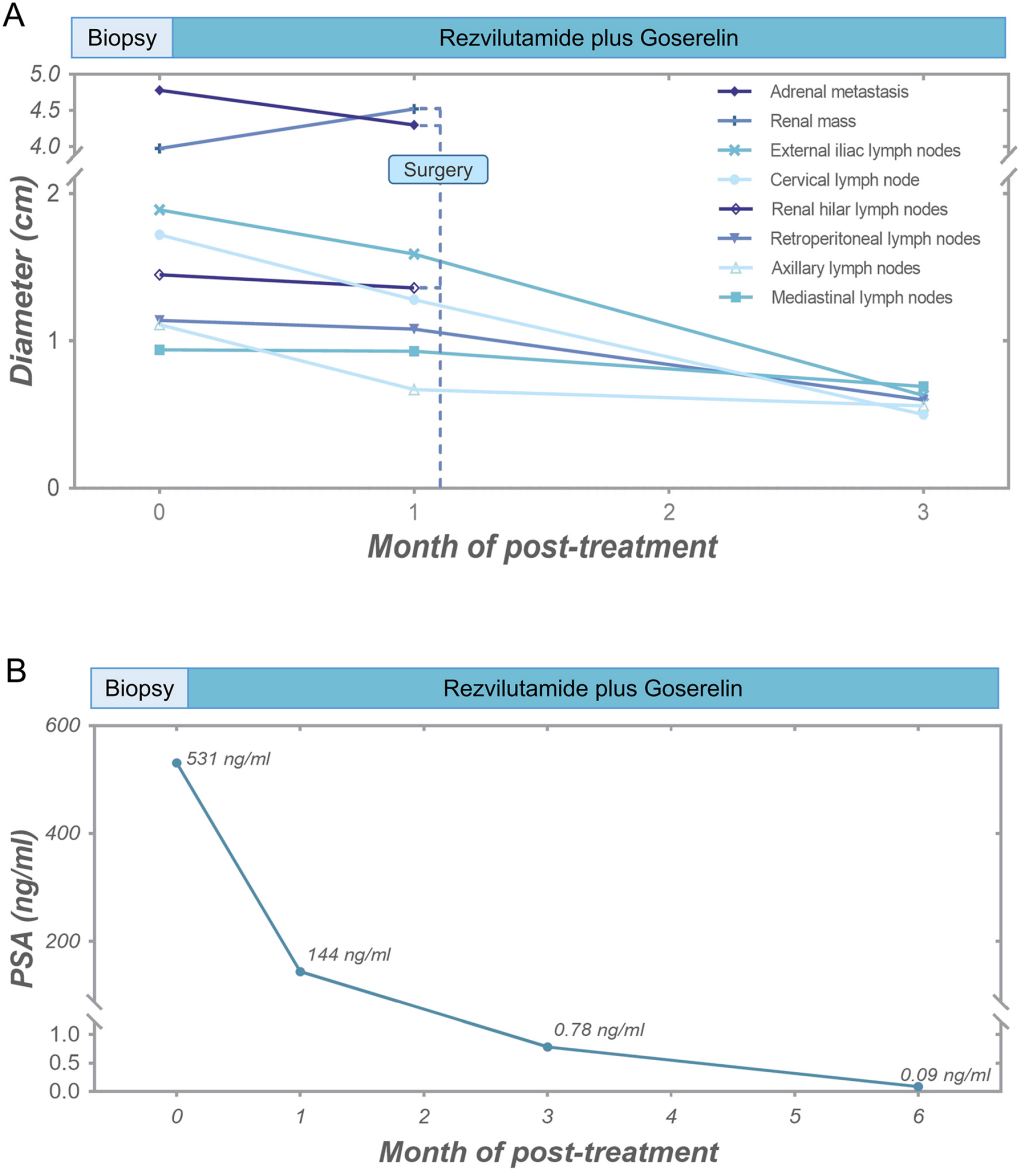
Tumor response of the patient. **(A)** Diameter alterations of tumors following anti-prostate cancer therapy and surgical procedure. **(B)** Post-treatment reductions in serum PSA levels.

Given the diagnosis of high-risk metastatic hormone-sensitive prostate cancer (mHSPC), the patient was treated with rezvilutamide and goserelin acetate sustained-release depot combination therapy. The CT scans performed at one and three months after treatment showed that all metastatic lesions were shrinking, with no signs of recurrence of RCC (**Figure 2A**; **Supplementary Figure 2**). Follow-up PSA levels, measured at one, three, and six months after treatment, were 144 ng/ml, 0.78 ng/ml, and 0.09 ng/ml, respectively (**Figure 2B**). Additionally, radionuclide bone scintigraphy did not detect any new lesions. The therapeutic outcomes substantiated that all metastatic lesions demonstrated a significant response to hormonal therapy.

## 3 Discussion

In this case report, we described for the first time a patient with concurrent PCa and RCC in whom the adrenal, renal hilar, and retroperitoneal lymph nodes, as well as other metastatic sites, were derived from PCa, not RCC. The patient was ultimately diagnosed with localized RCC and high-risk mHSPC with metastases to the left adrenal gland, bones, and multiple distant lymph nodes.

The synchronous occurrence of RCC and PCa is uncommon. Data from previous studies showed that the incidence of patients diagnosed with both PCa and RCC ranged from 0.83% to 2.10%, while the rate of synchronous occurrence was even lower (7–10). Meanwhile, metastasis from prostate cancer to the kidney has also been observed in case reports (11, 12), which is extremely rare but actually exists. Furthermore, two cases of retroperitoneal lymph node metastases containing both RCC and PCa, a phenomenon known as ‘collision metastasis’, have been reported in previous studies (13, 14). Therefore, the co-existence of RCC and PCa presents a complex diagnostic and therapeutic challenge. Lymph nodes are a common site of metastasis for both PCa and RCC, but the involvement of retroperitoneal lymph nodes is an uncommon presentation in PCa (15). Metastases to the perirenal tissues are exceedingly rare (16, 17). However, in patients with concurrent RCC and PCa, the retroperitoneal lymph node metastases may not always originate from the RCC. Furthermore, although the incidence of adrenal metastasis in metastatic RCC (8.9%, 991/11157) (6) is higher than that in metastatic PCa (1.9%, 12/620) (18), both possibilities remain. Therefore, for patients presenting with synchronous RCC and PCa, biopsies are essential for determining the origin of metastatic lesions.

Previously, 16 cases of adrenal metastasis from PCa have been reported (19–32), which were summarized in **Table 2**. All patients had high Gleason scores. Adrenal metastases from prostate cancer occurred predominantly in patients with mCRPC, and only 2 patients reported adrenal metastases in patients with mHSPC (24, 26). In the reported cases, common specific symptoms were absent, though abdominal pain could be a possible exception in cases of large tumors. More than half of the patients (10/16) underwent adrenalectomy, with the majority experienced disease remission after surgery, especially those with isolated adrenal metastases. Among these cases, no patients had concomitant renal mass or renal hilar lymph node metastasis.

**Table 2:** Summary of reported prostate cancer cases with adrenal metastases.

Study	Initial characteristics								Post-adrenal metastasis discovery							
	Age	Symptoms	Gleason score	PSA (ng/ml)	Tumor type	Surgery	Radiation therapy	Other treatments for mPCa	PSA (ng/ml)	Stages of patients	Site of adrenal metastases	Maximum diameter of adrenal metastases	Other sites of metastasis	Treatment for mPCa	Special treatment for adrenal metastases	Outcome
Topal 2024 (19)	66	NA	NA	NA	NA	NA	NA	Chemotherapy (docetaxel)	61.9	mCRPC	Bilateral	NA	No	NA	4 cycles (4 × 7.4 Gbq) of ^177^Lu-PSMA-617 at 6–8 weeks intervals	Post-treatment PSA levels dropped to 0.3 ng/mL and further decreased to 0.084 ng/mL at 6 months.
Sakellakis, 2023 (20)	60	No symptoms	5+4 = 9	NA	Adenocarcinoma	RP with LND	Yes	Leuprolide	63.6	mCRPC	Left	5.0 cm	No	Leuprolide	Left adrenalectomy	Nadir PSA was 0.7 ng/mL post-operatively, but it eventually increased to 28.5 ng/mL, with a local recurrence in the left suprarenal area within 21 months.
	70	Intermittent hematuria	4+4 = 8	9.4	Adenocarcinoma	No	No	Leuprolide	0.6	mCRPC	Right	2.7 cm	No	Leuprolide	Right adrenalectomy	PSA remained undetectable for 15 months.
	60	No symptoms	3+4 = 7	6.7	Adenocarcinoma	RP	Yes	Leuprolide; Bicalutamide; Abiraterone acetate plus prednisone	NA	mCRPC	Right	NA	Bone; Retroperitoneal LN	Enzalutamide; Diethylstilbesterol	Right adrenalectomy	PSA increased after 5 months.
Kanatsız 2023 (21)	61	NA	4+5 = 9	NA	Adenocarcinoma	RP	Yes	Chemotherapy; ADT	NA	mCRPC	Bilateral	Right: 4.0 cm Left: 3.2 cm	No	NA	NA	NA
Soriano 2022 (22)	86	NA	8	NA	mPCa with hepatoid differentiation	RP	Yes	Goserelin; Bicalutamide; Abiraterone and zolendronic; Enzalutamide	16.11	mCRPC	Left	2.9 cm	Bone; Retrocrural and retroperitoneal LN	Chemotherapy (cabazitaxel)	No	NA
Zhao 2022 (23)	65	NA	4+5 = 9	387.2	Adenocarcinoma	RP with LND	Yes	ADT	54.7	mCRPC	Bilateral	NA	No	Chemotherapy (docetaxel) and ADT	No	PSA level decreased to 6.6 ng/mL after 7 courses of docetaxel, and the bilateral adrenal masses reduced within 9 months.
Ribeiro 2022 (24)	68	NA	4+5 = 9	NA	Adenocarcinoma	RP	Yes	No	1.97	mHSPC	Left	NA	No	Systemic treatment	No	PSA level decreased significantly.
Muñoz López 2022 (25)	68	Acute urinary retention	NA	3.7	Neuroendocrine carcinoma	Bipolar transurethral resection	No	ADT plus chemotherapy	NA	mCRPC	Bilateral	Right: 12 cm Left: 12.5 cm	No	NA	Bilateral adrenalectomy	The patient remains alive with no reported deterioration during follow-up.
McGeorge 2021 (26)	79	NA	4+5 = 9	NA	NA	No	Yes	No	5.2	mHSPC	Right	2.2 cm	Lung	Goserelin	No	Confirmed castrate-resistance
									↓ (ADT) ↓						↓	↓
									18.0	mCRPC	Right	4.3 cm	No		Right adrenalectomy	PSA initially dropped to 0.09 ng/ml after surgery but rose to 1.6 ng/mL within 12 months, with metastases to para-aortic and inferior mediastinal lymph nodes.
Ashrafi 2020 (27)	77	Vague abdominal discomfort	NA	11	Adenocarcinoma	RP	NA	ADT	1.0	mCRPC	Left	5.9 cm	No	ADT	Left adrenalectomy	At 5 years follow-up, PSA levels remained undetectable.
Matrone 2015 (28)	69	NA	3+5 = 8	459.6	NA	No	Yes	ADT	106.8	mCRPC	Right	3.5 cm	External iliac LN	Triptorelin	Right adrenalectomy	Post-treatment PSA levels dropped to 0.76 ng/mL and further decreased to 0.01 ng/mL at 10 months.
Subhawong 2010 (29)	71	NA	5+5 = 10	23	Adenocarcinoma	No	Yes	ADT; Anti-androgens; Ketoconazole; Hydrocortisone	27.0	mCRPC	Left	12.0 cm	Retroperitoneal LN	No	Left adrenalectomy	After surgery, the patient's PSA declined to normal range without requiring adjuvant therapy.
Barrisford 2009 (30)	39	Lower back pain	4+5 = 9	40	Adenocarcinoma	RP and LND	Yes	Neoadjuvant leuprolide; Leuprolide acetate and bicalutamide; Ketoconazole	151.4	mCRPC	Left	6.8 cm	No	Leuprolide	Left adrenalectomy (only resect the bulk of the tumor)	The PSA was 17.8 ng/mL one month postoperative, but began to rise at the sixth week.
Kawahara 2009 (31)	65	Increased urinary frequency	4+3 = 7	515	NA	No	No	Maximum androgen blockade; Dexamethasone plus docetaxel	NA	mCRPC	Bilateral	NA	Bone; Pleural	NA	NA	NA
Sakamoto 1999 (32)	64	NA	NA	1020	Adenocarcinoma	No	No	ADT	3.3	mCRPC	Left	2 cm	Bone; Lung	Adjuvant chemotherapy (vinblastine and estramustine); LH-RH agonist plus UFT	Left adrenalectomy	After surgery, PSA levels dropped below 0.1 ng/mL, and no disease progression was observed with LH-RH agonist and UFT treatment.

PSA, prostate-specific antigen; NA, not available; RP, radical prostatectomy; LND, lymph node dissection; mPCa, metastatic prostate cancer; mCRPC, metastatic castration-resistant prostate cancer; mHSPC, metastatic hormone-sensitive prostate cancer; ADT, androgen deprivation therapy; LN, lymph node; LH-RH agonist, luteinizing hormone-releasing hormone agonist; UFT, mixture of tegafur and uracil. ↓, changes in the disease status, treatment plan, and clinical outcomes.

DRE and PSA screening are the most common prostate cancer screening methods. Despite PSA screening can lead to overdiagnosis and potential overtreatment in early low-risk prostate cancer, PSA screening remains crucial (33). Previous studies have revealed a low coverage of PSA screening in most Asian countries (34). This patient, for instance, had never undergone PSA screening or a DRE, presenting with widespread metastatic disease at initial diagnosis, which underscores the critical nature of prostate cancer screening.

The treatment methods for metastatic RCC and metastatic PCa are distinct. The common first-line treatments for advanced or metastatic RCC include single-agent targeted therapy or immune checkpoint inhibitor (ICI)-based combination therapy (35). In contrast, the treatment approaches for high-risk mHSPC typically involve combination therapies based on androgen deprivation therapy (ADT) (36). Hence, the accurate staging of both RCC and PCa as well as the identification of the primary tumor is of great importance. In this case, the RCC is small with the ISUP grade of 3, while the perinephric lymph nodes and adrenal metastases all originating from the prostate. Consequently, the patient was diagnosed with localized RCC coexisting with high-risk mHSPC. After receiving combination therapy based on ADT, there was a significant reduction in all metastatic lesions, resulting in stable disease management. Therefore, in cases with multiple primary malignancies, the accurate staging of different tumors is crucial for the success of the treatment.

## 4 Conclusion

In this case, conventional radiological and nuclear medicine examinations proved insufficient to determine the primary site of the tumor and accurately stage the malignancies. Percutaneous biopsies confirmed the presence of two synchronous tumors. Subsequent pathological analysis of the surgically removed tissues identified the origin of the metastasis and accurately staged the patient’s renal and prostate cancers, leading to a more favorable therapeutic response. This case highlights that metastatic lesions in the adrenal gland and renal hilar lymph nodes may not exclusively originate from the RCC but could also arise from the PCa in the context of concurrent RCC and PCa.

## Data Availability

The original contributions presented in the study are included in the article/**Supplementary Material**. Further inquiries can be directed to the corresponding author.
