# Supplementary material for: Case report: Synchronous prostate cancer and renal cell carcinoma with prostate cancer-origin metastases to adrenal and renal hilar lymph nodes
Source: Front Oncol. 2024 Oct 7;14:1412067. doi: 10.3389/fonc.2024.1412067 (PMC11491287; doi:10.3389/fonc.2024.1412067)
Supplement: Supplementary file 1 [file DataSheet1.pdf]

Supplementary Material

Supplementary Figures:

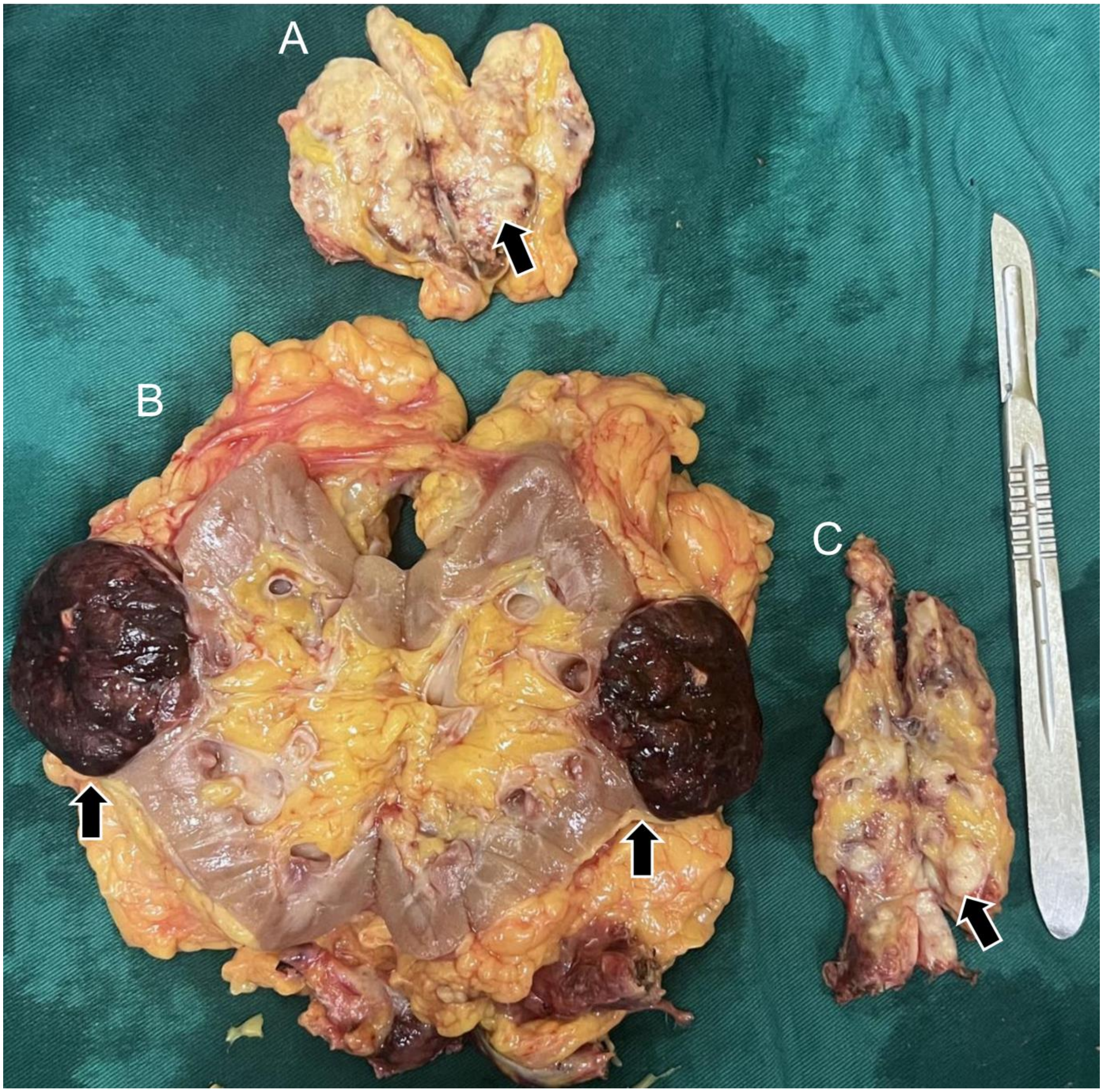

Supplementary Figure 1. Gross surgical resection specimen. (A) Left adrenal gland and metastatic tumor (black arrow); (B) Left kidney and tumor (black arrows); (C) Retroperitoneal lymph nodes including renal hilar lymph nodes (black arrow);

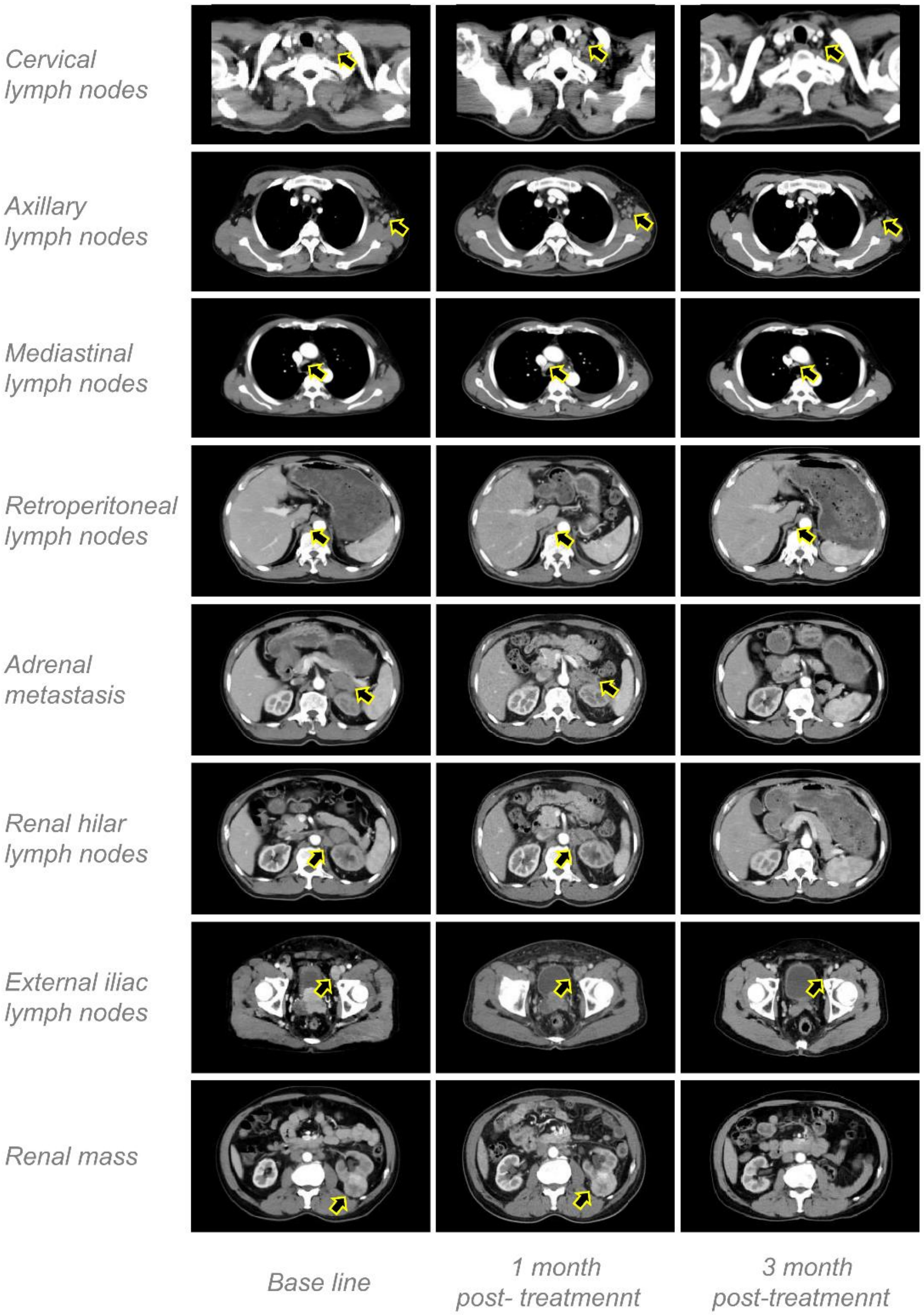

Supplementary Figure 2. Tumor response based on computed tomography scans of different lesions.
